# Cost-effectiveness of antibiotic treatment of uncomplicated urinary tract infection in women: a comparison of four antibiotics

**DOI:** 10.3399/bjgpopen17X101097

**Published:** 2017-10-04

**Authors:** Susannah Sadler, Michael Holmes, Shijie Ren, Stephen Holden, Swati Jha, Praveen Thokala

**Affiliations:** 1 Research Associate, School of Health and Related Research, University of Sheffield, Sheffield, UK; 2 Research Fellow, University of Exeter Medical School, University of Exeter, Exeter, UK; 3 Research Associate, School of Health and Related Research, University of Sheffield, Sheffield, UK; 4 Research Associate, School of Health and Related Research, University of Sheffield, Sheffield, UK; 5 Consultant Microbiologist, Department of Medical Microbiology, Nottingham University Hospitals NHS Trust, QMC Campus, Nottingham, UK; 6 Consultant Gynaecologist, Gynaecology, Sheffield Teaching Hospitals NHS Foundation Trust, Sheffield, UK; 7 Research Fellow, School of Health and Related Research, University of Sheffield, Sheffield, UK

**Keywords:** urinary tract infection, antibiotic, cost-effectiveness, resistance, primary care

## Abstract

**Background:**

Urinary tract infections (UTIs) are one of the most common reasons for women to attend primary care. There are four different antibiotics currently recommended in England for treatment of uncomplicated UTI but little evidence on their comparative cost-effectiveness.

**Aim:**

To assess the relative cost-effectiveness of the four antibiotics currently recommended in England for treatment of uncomplicated UTI in adult women.

**Design & setting:**

A cost-effectiveness model in adult women with signs and symptoms of uncomplicated UTI in primary care in England treated with fosfomycin, nitrofurantoin, pivmecillinam, or trimethoprim.

**Method:**

A decision tree economic model of the treatment pathway encompassed up to two rounds of treatment, accounting for different resistance levels. End points included recovery, persistence, pyelonephritis, and/or hospitalisation. Prescription, primary and secondary care treatment, and diagnostic testing costs were aggregated. Cost-effectiveness was assessed as cost per UTI resolved.

**Results:**

Trimethoprim 200 mg twice daily (for 3 or 7 days) was estimated to be the most cost-effective treatment (£70 per UTI resolved) when resistance was <30%. However, if resistance to trimethoprim was ≥30%, fosfomycin 3 g once became more cost-effective; at resistance levels of ≥35% for trimethoprim, both fosfomycin 3 g once and nitrofurantoin 100 mg twice daily for 7 days were shown to be more cost-effective.

**Conclusion:**

Knowing local resistance levels is key to effective and cost-effective empirical prescribing. Recent estimates of trimethoprim resistance rates are close to 50%, in which case a single 3 g dose of fosfomycin is likely to be the most cost-effective treatment option.

## How this fits in

Four different antibiotics are currently recommended for treatment of uncomplicated urinary tract infection (UTI) in adult women in England. It is usual practice to treat empirically at first presentation, but no studies to date have compared the relative cost-effectiveness of these treatments, so there is little to guide clinicians in their prescribing choice. The results of this study will help guide clinicians faced with decisions about empirical prescribing, to choose the most cost-effective option, especially in the context of local knowledge of resistance levels.

## Introduction

UTIs are one of the most common reasons for women to attend primary care, and are likely to affect at least half of all women in their lifetime.^1^ In England in 2011, 14% of antibiotic prescriptions for community-acquired infections were for UTI.^2^ Nitrofurantoin, a recommended first-line UTI treatment in England with no other recommended use, was prescribed more than 2.3 million times in 2015.^3^

For women with suspected uncomplicated UTI, Public Health England (PHE) recommends first-line treatment with nitrofurantoin, trimethoprim, or pivmecillinam. Fosfomycin or pivmecillinam are indicated where resistance risk is higher.^4^ In most cases, empirical treatment without urine culture is recommended and, as a result, the causative organism and its antimicrobial susceptibility are unknown. In practice, trimethoprim prescribing is still very common, despite some evidence of high levels of resistance. Although nitrofurantoin prescribing is still increasing,^5^ actual prescribing practice varies considerably between local areas.^6^

Antibiotic resistance is a key threat to public health; good prescribing practice is essential to reduce the spread of resistance.^6^ The aims of antibiotic prescribing should be to ensure treatment is effective, while minimising cost and reducing 'collateral damage' such as the emergence of multidrug resistant pathogens. As such, a good understanding of the effectiveness and cost-effectiveness of the drug, as well as national and local resistance levels, are necessary to aid decision making in primary care.

For clinical decision making, in which several relevant treatments options are recommended, it is important to understand the comparative efficacy and cost-effectiveness of all options. Although clinical trials to date have made direct comparisons between treatments, network meta-analyses (NMAs — allowing direct and indirect treatment comparisons) are needed in order to understand how the different treatments compare. Two previous meta-analyses of treatments in uncomplicated UTI have been undertaken,^7,8^ but neither includes clinical outcomes for all the treatments currently recommended by PHE for uncomplicated UTI in England, and neither extends its findings to cost-effectiveness analysis. This study aimed to compare the effectiveness and cost-effectiveness of these treatments and to explore the effect of changing resistance levels to trimethoprim.

## Method

### Model structure

**Figure 1.**
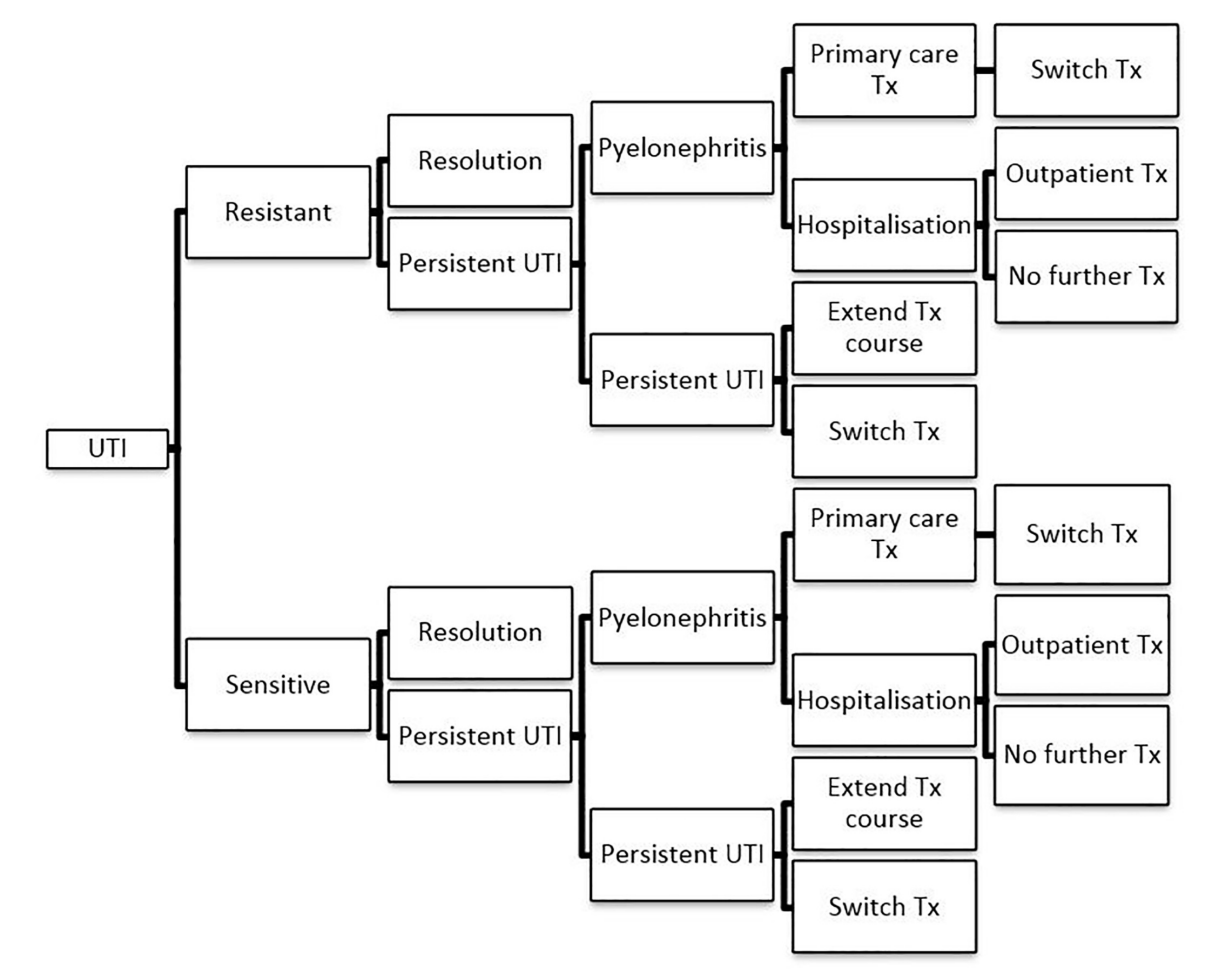
Model pathway. Tx = treatment. UTI = urinary tract infection.

The model was set in the context of the NHS in England. It was based on a decision tree model (Figure 1) developed by McKinnell *et al,* ^9^ updated to include UK-specific costs. The pathway was checked by specialist clinicians.

In the model, patients were prescribed an antibiotic treatment regimen at their first GP appointment. The infection responded or failed to respond to treatment, depending on whether bacteria were resistant or susceptible to the antibiotic. Persistence of symptoms resulted in a repeat GP visit and second prescription. A potential consequence of persistent infection was pyelonephritis, treated either in hospital or primary care, in line with UK practice. It was assumed that all patients treated in hospital had a follow-up outpatient visit, and that all patients treated for a second time in primary care for either persistent UTI or pyelonephritis switched to a different antibiotic for their second course of treatment, in line with PHE guidance.^4^

Model timings were 9 days for the initial treatment round, followed by the weighted average of follow-up periods in the trials used for effectiveness data:

- 7days for second-round treatment if in primary care; or
- 5 days if in hospital (based on a recent UK study)^10^ plus 2 days of outpatient treatment for pyelonephritis.

This gave a total of 16 days. After two treatment courses all patients were assumed to have achieved cure.

### Clinical effectiveness

**Figure 2.**
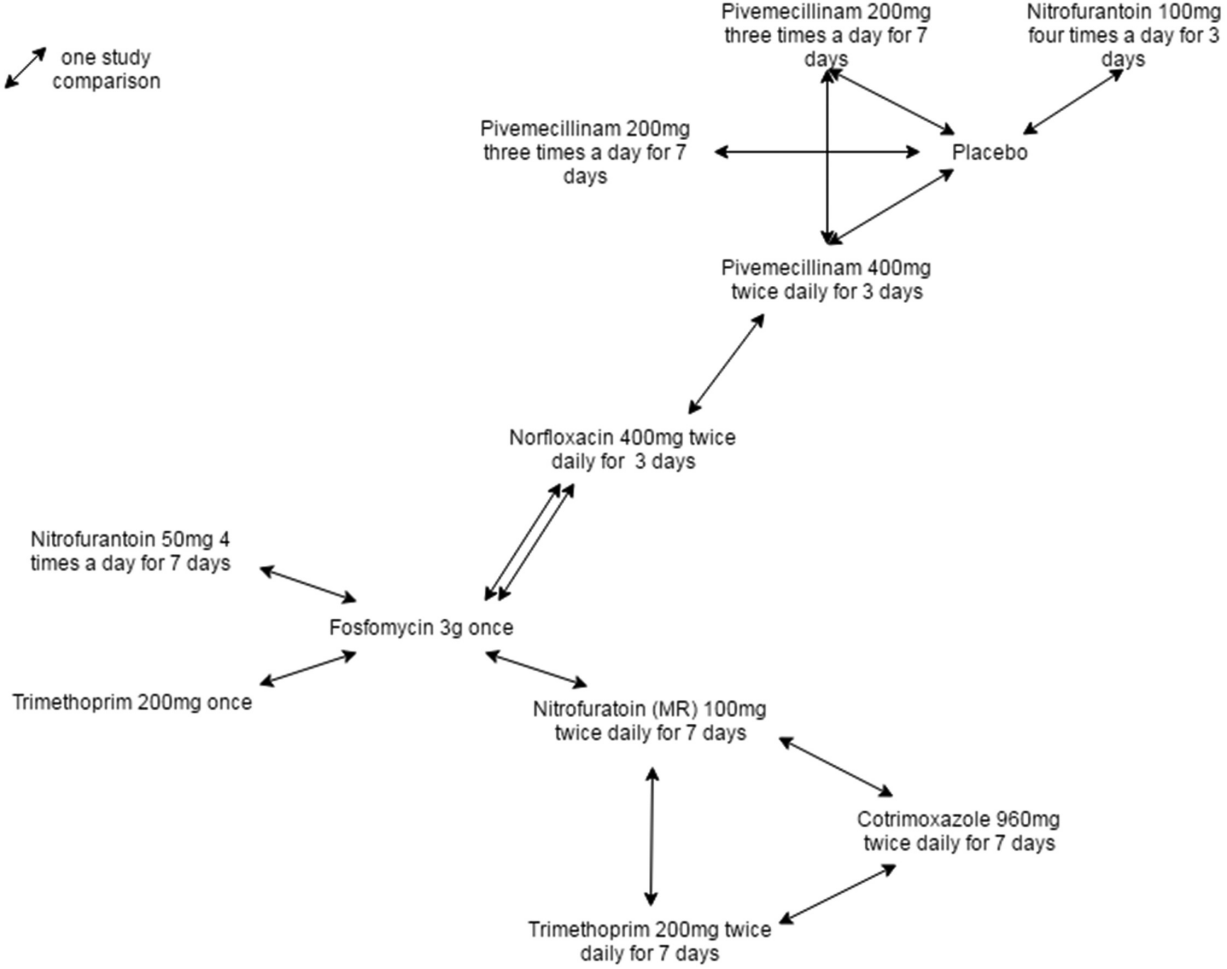
Network of trials identified in the systematic review and included in the network meta-analysis to estimate relative effectiveness of different treatment regimens.

Clinical cure rates were informed by a systematic review and NMA of studies in adult women with signs and symptoms of uncomplicated UTI (Appendix 1). The systematic review identified 11 studies that formed a connected evidence network used in the NMA (Figure 2).^11–21^ The studies covered nine treatment regimens:

- nitrofurantoin 50 mg four times a day for 7 days;
- nitrofurantoin modified release (MR) 100 mg twice daily for 7 days;
- nitrofurantoin 100 mg four times a day for 3 days;
- pivmecillinam 200 mg three times a day for 7 days;
- pivmecillinam 400 mg twice daily for 3 days;
- pivmecillinam 200 mg twice daily for 7 days;
- trimethoprim 200 mg twice daily for 7 days;
- trimethoprim 200 mg once; and
- fosfomycin 3 g once.

A random (treatment) effects model with a logit link function was used to allow for heterogeneity in treatment effects between studies. The model assumed a fixed (that is, unconstrained) baseline effect in each study so that treatment effects were estimated within study and combined across studies. The NMA model fitted the data well, with a total residual deviance of 20.47 being close to the number of data points included in the analysis (*n *= 21). The between-study standard deviation was estimated to be 0.21 (95% credible interval = 0.01 to 0.68), implying mild heterogeneity in treatment effects between studies. Clinical cure rates for each of these regimens derived from the NMA are reported in Table 1.

**Table 1.** Results of the network meta-analysis.

	Results of NMA of 11 RCTs			**Model parameters derived from NMA using McNulty *et al*** ^**22**^		**Resistance rate from ECO-SENS II,** ^**23**^ ** %**
	Odds ratio	95% CI	Posterior mean cure rate, %	Resistant cure rate, %	Sensitive cure rate, %	
Fosfomycin 3 g once	1	–	84.2	53.1	84.3	0.5
Nitrofurantoin 50 mg four times a day for 7 days	0.82	0.30 to 2.18	79.9	50.3	79.9	0.0
Nitrofurantoin (MR) 100 mg twice daily for 7 days	1.15	0.55 to 2.56	85.0	53.6	85.0	0.0
Nitrofurantoin 100 mg four times a day for 3 days	0.34	0.06 to 2.05	62.4	39.3	62.4	0.0
Pivmecillinam 200 mg three times a day for 7 days	0.63	0.16 to 2.57	75.0	47.4	75.3	1.0
Pivmecillinam 400 mg twice daily for 3 days	0.47	0.14 to 1.57	69.8	44.2	70.1	1.0
Pivmecillinam 200 mg twice daily for 7 days	0.69	0.17 to 2.78	76.2	48.2	76.5	1.0
Trimethoprim 200 mg twice daily for 7 days	1.29	0.43 to 4.02	85.9	57.3	90.8	14.9
Trimethoprim 200 mg once	0.31	0.06 to 1.38	61.1	40.8	64.7	14.9

None of the treatment effects were statistically significantly different at a conventional 5% level, and pairwise comparisons indicated that no one treatment was significantly more effective than any other. CI = credible interval. MR = modified release. NMA = network meta analyses. RCT = randomised controlled trial.

The ratio of resistant to sensitive cure rate (0.63) was applied to overall cure rates from the NMA to estimate sensitive and resistant cure rates for each regimen. This ratio was taken from a UK prospective cohort study that found statistically significant differences in clinical cure rates between those infected with trimethoprim-resistant and susceptible organisms,^22^ and was assumed to be consistent across all treatments. Resistance rates to each drug were taken from the ECO-SENS II study,^23^ which provided UK-specific resistance rates for *Escherichia coli* only. Table 1 summarises the cure rates and resistance rates used in the base case.

#### Other model parameters

The GP appointment cost was taken from the Personal Social Services Research Unit's *Unit Costs of Health and Social Care 2014*.^24 ^The dipstick test cost was taken from a Health Technology Assessment by Little *et al,* ^25 ^and the Healthcare Resource Groups' national schedule of reference costs^26^ was used for the cost of pyelonephritis hospitalisation, pyelonephritis outpatient visits, and urine analysis tests. The cost of nitrofurantoin, trimethoprim, and pivmecillinam were taken from the *British National Formulary,* ^27 ^whereas the cost of fosfomycin was provided by the manufacturer since it is not listed in the *BNF*.

As in McKinnell *et al*,^9^ the authors assumed that 4% of those not achieving clinical cure at first treatment develop pyelonephritis, and 20% of those with pyelonephritis require hospitalisation. Model parameters are summarised in Table 2.

**Table 2.** Model parameters, including costs and treatment pathways, used in the model

Parameter	Type	Mean cost, £	Source
Fosfomycin 3 g once	Prescription	4.86	Profile Pharma^a^
Nitrofurantoin 50 mg four times a day for 7 days	Prescription	13.93	*BNF* ^27^
Nitrofurantoin (MR) 100 mg twice daily for 7 days	Prescription	9.50	
Nitrofurantoin 100 mg four times a day for 3 days	Prescription	8.14	
Pivmecillinam 200 mg three times a day for 7 days	Prescription	9.45	
Pivmecillinam 400 mg twice daily for 3 days	Prescription	5.40	
Pivmecillinam, 200 mg twice daily for 7 days	Prescription	6.30	
Trimethoprim 200 mg twice daily for 7 days	Prescription	1.00	
Trimethoprim 200 mg once	Prescription	0.07	
Pyelonephritis	Hospitalisation	3992.00	National schedule of reference costs^26^
Pyelonephritis	Outpatient visit	94.00	
Urine analysis	Test	7.00	
GP appointment	Per patient contact (11.7 minutes)	46.00	PSSRU^24^
Dipstick test	Test	0.40	Little *et al* ^25^
Pathway			
Risk of pyelonephritis if clinical cure not achieved, %		4.00	McKinnel *et al* ^9^
Risk of hospitalisation if pyelonephritis, %		20.00	

^a^This price was provided by Profile Pharma, which is the approved UK distributor of Monuril (fosfomycin trometamol) on behalf of the marketing authorisation holder Zambon. Monuril was launched onto the UK market at this price in August 2016. BNF = British National Formulary. MR = modified release. PSSRU = Personal Social Services Research Unit.

#### Analysis

The outcome was cost per UTI resolved. No incremental analysis was carried out as all treatments assessed are currently recommended for use in the NHS in England.

### Sensitivity analysis

To account for uncertainty, probabilistic sensitivity analysis (PSA) was carried out using 2000 sets of model results. Parameters were sampled from the following distributions:

- beta (resistance rates);
- gamma (health service costs); and
- the posterior distribution of the NMA (clinical cure rates).

Resistance to nitrofurantoin (0%) and prescription costs were fixed. PSA results were illustrated on a cost-effectiveness plane.

Deterministic analyses were carried out to test the sensitivity of model outcomes to the following.

#### Incorporation of resistance rates to bacteria other than *E. coli* 

This was done because nitrofurantoin — despite having 0% resistance rate for *E. coli *— is non-effective against some strains of *Klebsiella* and *Enterobacter*, and most strains of *Proteus*. Resistance rates were estimated using the distribution of bacterial isolates in uncomplicated UTI for the UK and Ireland taken from an earlier ECO-SENS report,^28 ^combined with (non-UK-specific) resistance rates to each of these pathogens from the original ECO-SENS results^29^), and for *E. coli* from the ECO-SENS-II results.^23^

#### Updated estimates of *E. coli* resistance 

These were recently published by Kahlmeter *et al,* ^30^ (trimethoprim: 46.0%, nitrofurantoin: 5.6%, and pivmecillinam: 4.8%). Results are from a single centre but suggest that resistance to trimethoprim is increasing.

#### Estimated cure rates for 3-day regimens for trimethoprim and nitrofurantoin 

This was done because 3-day courses of trimethoprim and nitrofurantoin are recommended by PHE, whereas 7-day regimens are reported in the randomised controlled trials (RCTs). The relative risk of treatment failure from Goettsch *et al* ^31^ between 3- and 7-day trimethoprim (0.87) and nitrofurantoin (0.64) regimens. Prescription costs were reduced accordingly.

In addition a threshold analysis was carried out; this varied the level of trimethoprim resistance between 15% and 50% in increments of 5% to determine whether the choice of the most cost-effective treatment regimen is affected by increasing levels of trimethoprim resistance.

## Results

### Probabilistic economic model results

Central estimates from the PSA in terms of costs, health outcomes, and cost per UTI resolved are reported in Table 3. Trimethoprim 200 mg twice daily for 7 days was estimated to be the most cost-effective treatment regimen at £70 per UTI resolved, followed by fosfomycin 3 g once at £78 per UTI resolved. Trimethoprim 200 mg twice daily for 7 days also had the highest probability of being the most cost-effective treatment (59% of PSA runs).

**Table 3.** Probabilistic costs, health outcomes, and cost per UTI resolved per treatment regimen modelled

	Total cost, £	UTIs resolved, *n*	Cost per UTI resolved, £				
			Baseline sensitivity analyses		Scenarios		
			Probabilistic	Deterministic	A	B	C
Trimethoprim 200 mg twice daily for 7 days	60	857	70	69	69	91	73
Fosfomycin 3 g once	65	842	78	77	80	77	
Nitrofurantoin (MR) 100 mg twice daily for 7 days	69	849	82	81	87	84	100
Pivmecillinam 200 mg twice daily for 7 days	67	766	88	96	98	99	
Nitrofurantoin 50 mg four times a day for 7 days	78	799	98	97	104	101	
Pivmecillinam 200 mg three times a day for 7 days	78	753	103	103	106	106	
Pivmecillinam 400 mg twice daily for 3 days	78	704	111	111	114	114	
Trimethoprim 200 mg once	81	609	133	131	130	161	
Nitrofurantoin 100 mg four times a day for 3 days	87	631	138	139	147	144	

Note: treatments ordered by lowest cost per UTI resolved.

Baseline analysis compared with the two deterministic scenarios tested: A = resistance rates from pathogens other than *E. coli;* B = updated resistance measures for trimethoprim, nitrofurantoin, and pivmecillinam; and C = estimated effectiveness for 3-day dosing, which is in line with the current guidance from Public Health England for use of these treatments. MR = modified release. UTI = urinary tract infection.

**Figure 3.**
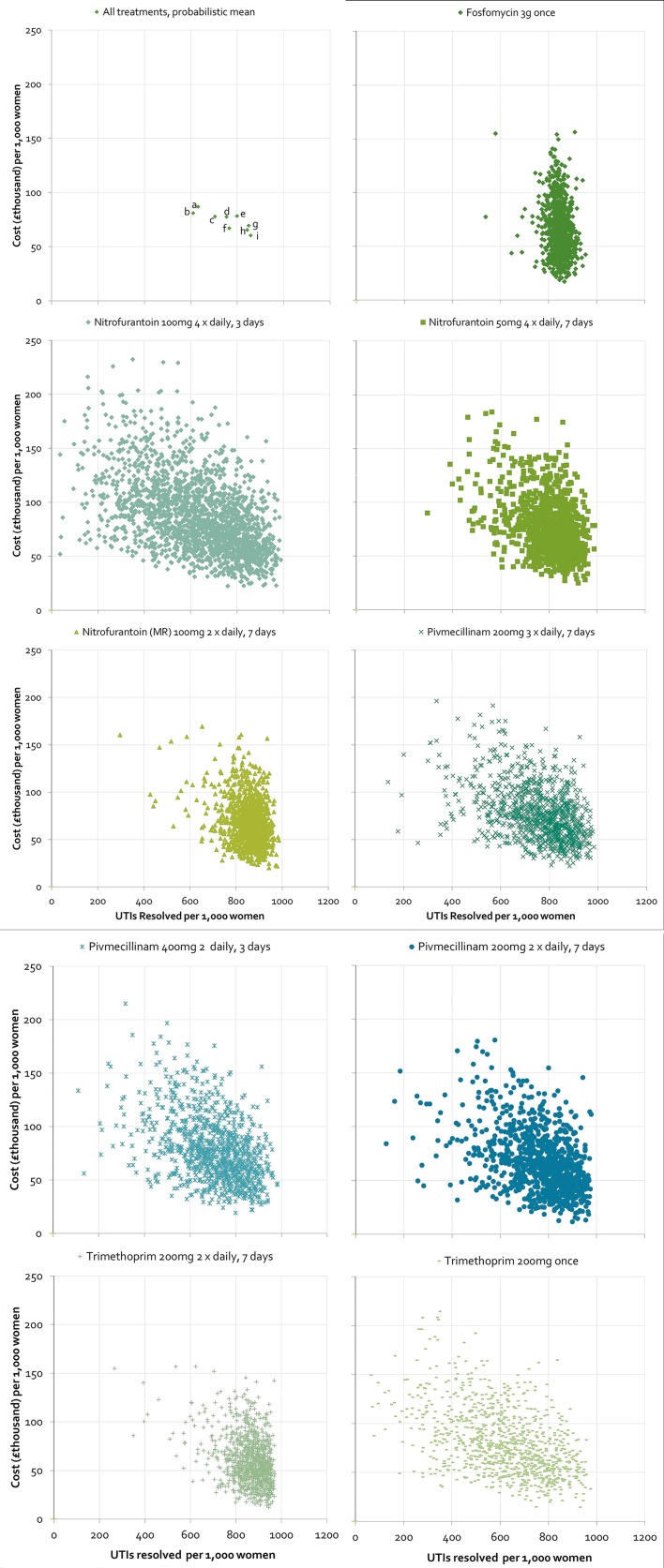
Cost-effectiveness plane comparing all treatment regimens.

Figure 3 shows the probabilistic average total cost and number of UTIs resolved per 1000 patients for each treatment regimen. A group of three treatments — trimethoprim 200 mg twice daily for 7 days, fosfomycin 3 g once, and nitrofurantoin 100 mg twice daily for 7 days — stood out as being most effective for resolution (approximately 850 cases resolved per 1000) and had the lowest total cost (£60 000–£70 000). Figure 3 illustrate the uncertainty around the central estimates of cost-effectiveness, showing the results of each of the 2000 probabilistic model runs for each treatment regimen.

### Deterministic sensitivity analysis

Results are summarised in Table 3. Scenario A had the expected effect of reducing the apparent cost-effectiveness of nitrofurantoin relative to the other treatments. Scenario B reduced the cost-effectiveness for all treatments with increased resistance, as expected. In particular, the cost-effectiveness of trimethoprim reduced significantly: from a deterministic value of £69 to £91 per UTI resolved for trimethoprim 200 mg twice daily for 7 days: this resulted in trimethoprim 200 mg twice daily no longer being the most cost-effective treatment. Scenario C reduced the cost-effectiveness of trimethoprim 200 mg twice daily for 7 days and nitrofurantoin (MR) 100 mg twice for 7 days, but trimethoprim 200 mg twice daily was still shown to be the most cost-effective treatment (up from £69 to £73 per UTI resolved against a cost of £100 per UTI resolved for nitrofurantoin).

The threshold analysis on trimethoprim resistance showed that for resistance of >25%, trimethoprim 200 mg twice daily for 7 days remained the most cost-effective option. However, at 30% resistance, fosfomycin 3 g once became more cost effective, and at resistance levels of ≥35% both fosfomycin 3 g once and nitrofurantoin (MR) 100 mg twice daily for 7 days appeared to be more cost effective than trimethoprim 200 mg twice daily.

## Discussion

### Summary

The highest clinical cure rate was estimated to be with trimethoprim 200 mg twice daily for 7 days. In general, higher cure rates were seen with 7-day regimens compared with 3-day regimens, however, treatment effects were not statistically significantly different.

Trimethoprim 200 mg twice daily for 7 days was estimated to be the most cost-effective treatment regimen, followed by fosfomycin 3 g once. In line with best practice for antimicrobial stewardship, 7-day trimethoprim prescriptions are now falling, with almost 50% of prescriptions being for the recommended 3-day courses.^32^ Due to lack of trial evidence, the authors estimated the impact of reducing the course length of both trimethoprim and nitrofurantoin from 7 to 3 days. This did not alter the fact that trimethoprim 200 mg twice daily was the most cost-effective treatment but nitrofurantoin (MR) 100 mg twice daily became less cost-effective than both pivmecillinam 200 mg twice daily for 7 days and nitrofurantoin 50 mg four times a day for 7 days.

The base case model results account only for resistance to *E. coli.* However, other species are known to have higher levels of resistance to all the antibiotics assessed. In particular, nitrofurantoin is non-effective against a number of *Klebsiella*, *Enterobacter*, and *Proteus* strains. When the authors accounted for resistance to other species, the cost-effectiveness was reduced (especially for nitrofurantoin) but the ranking of treatments was unaffected.

Recent work points to considerable increases in the resistance of common uropathogens. Kahlmeter *et al* observed increased rates of resistance of *E. coli* in uncomplicated UTI in the UK to nitrofurantoin, pivmecillinam, and trimethoprim.^30^ At this higher level of resistance, and even at resistance levels as low as 30%, trimethoprim 200 mg twice daily was no longer the most cost-effective treatment. Assuming fosfomycin resistance is unchanged (to date, it has been rarely prescribed in the UK and there is some evidence that resistance rates — at least to *E. coli* — remain stable, even in countries with systematic fosfomycin use),^33^ fosfomycin 3 g once would be the most cost-effective option for empirical treatment, followed by nitrofurantoin (MR) 100 mg twice daily for 7 days.

### Strengths and limitations

There were several limitations in this analysis. There was a lack of evidence available to inform differential cure rates with resistant versus sensitive bacteria strains. Due to a lack of RCT evidence, the authors estimated the differential rates from the ratio of sensitive to resistant cure in a UK cohort study that investigated trimethoprim only,^11^ based on expert clinical opinion. The results of the study conformed to prior expectations: that is, that cure rates would be lower in matched patients infected with organisms resistant to the treatment antibiotic. The derivation also reflects the fact that clinical resolution occurs in a proportion of patients who were not treated (previous studies showed rates ranging from 25% to 42%),^12–14^ and that when patients are treated with an antimicrobial agent to which the infecting uropathogen is resistant on laboratory testing, it is generally expected that cure rates will be higher than with placebo.

The study design had a number of important strengths: the context was the English health service; laboratory testing and clinical management were in accordance with established practice and national recommendations that are still broadly the same at present; and patients with host factors that could bias the data, such as structural abnormalities of the renal tract, pregnancy, and recurrent UTIs, were excluded.

### Comparison with existing literature

Le and Miller^34^ carried out a similar analysis in a US setting, comparing trimethoprim-sulfamethoxazole (TMP-SMX — the recommended first-line treatment for uncomplicated UTI in women) with fluoroquinolones (recommended when resistance levels are >10%); subsequently, McKinnel *et al* compared nitrofurantoin to these two treatments, also in a US setting: increasing TMP-SMX resistance was shown to increase mean costs of UTI treatment such that when resistance to TMP-SMX exceeded 22%, fluoroquinolones were the cheaper option,^34^ and that when fluoroquinolone resistance exceeded 12%, nitrofurantoin was the least costly option.^9^ Similarly, the study presented here showed trimethoprim to be the most cost-effective option compared with the other treatments recommended in England, as long as resistance was <30%.

### Implications for research and practice

Several pieces of additional evidence would enhance the authors' model estimates, were they available. Very few studies analysed both *in vitro* susceptibility and clinical response, meaning differential cure rates for sensitive and resistant strains had to be estimated. Similarly, recent, multicentre antimicrobial resistance surveillance data from all female patients with uncomplicated UTI, including those ordinarily treated empirically without sampling, would be very valuable.

Although this analysis confirmed that all four treatments that are currently recommended for uncomplicated UTI in England are effective in terms of efficacy and relative cost-effectiveness, trimethoprim 200 mg twice daily for either 3 or 7 days appeared to be the preferable treatment. However, evidence of rapid increases in trimethoprim resistance in the UK, coupled with the potential for local-level variation, casts doubt on its cost-effectiveness in empirical treatment of uncomplicated UTI. Assuming resistance to fosfomycin has not increased since 2008, fosfomycin 3 g once appears to be the most cost-effective option for empirical treatment given the potentially high levels of trimethoprim resistance.

The four drugs examined all have a relatively low propensity to cause *Clostridium difficile* infection and it is likely that acquired resistance to nitrofurantoin, pivmecillinam, and fosfomycin — despite widespread global use for many years — has not readily emerged due to their rapid absorption and minimal impact on the human gastrointestinal tract flora. These properties make them ideal treatments for uncomplicated UTI.

The modelling estimates in this study suggest that fosfomycin 3 g once is likely to be the most cost-effective choice for first-time empirical treatment of uncomplicated UTI in adult women, unless trimethoprim resistance is believed to be <30%; when resistance exceeds 35%, nitrofurantoin (m[MR]) 100 mg twice daily would also be a cost-effective choice.

## Appendix

**Appendix 1:**

Systematic review inclusion/exclusion criteria
Population: women aged >18 years with signs and symptoms of uncomplicated UTI
Interventions: fosfomycin, trimethoprim, nitrofurantoin, pivmecillinam (those recommended for treatment of uncomplicated UTI by Public Health England).
Outcomes: UTI resolution, persistence, pyelonephritis development and health-related quality of life (HRQoL)
Exclusion criteria: no clinical response measure, study not in English, studys specifically of older people patients who were pregnant or had a catheter
**Search strategy**
Randomised controlled trial (RCT) and systematic review study search strategies
Database: Ovid MEDLINE® In-Process & Other Non-Indexed Citations, Ovid MEDLINE® Daily, Ovid MEDLINE®, and Ovid OLDMEDLINE® <1946 to Present>
**Search strategy:**
* Population terms (1–6)*
exp Urinary Tract Infections/ urinary tract infection$.ab,ti. uti.ab,ti. acute cystitis.ab,ti. Cystitis/ 1 or 2 or 3 or 4 or 5 *Intervention terms (7–14)* Fosfomycin/ fosfomycin.ab,ti. phosphonomycin.ab,ti. phosphomycin.ab,ti. monuril.ab,ti. monurol.ab,ti. 2N81MY12TE.rn. 7 or 8 or 9 or 10 or 11 or 12 or 13 *Population and intervention terms combined (15)* 6 and 14 *Comparator terms (16–47)* Nitrofurantoin/ nitrofurantoin.ab,ti. furadoine.ab,ti. furantoin.ab,ti. macrodantin.ab,ti. furadonine.ab,ti. furadantine.ab,ti. furadantin.ab,ti. macrobid.ab,ti. 927AH8112L.rn. Trimethoprim/ trimethoprim.ab,ti. proloprim.ab,ti. trimpex.ab,ti. monotrim.ab,ti. triprim.ab,ti. tmi.ab,ti. tmp.ab,ti. AN164J8Y0X.rn. Amdinocillin Pivoxil/ pivmecillinam.ab,ti. amdinocillin.ab,ti. selexid.ab,ti. pivamdinocillin.ab,ti. fl 1039.ab,ti. fl-1039.ab,ti. fl1039.ab,ti. mecillinam.ab,ti. penomax.ab,ti. coactabs.ab,ti. 1WAM1OQ30B.rn. or/16-46 *Population and comparator terms combined (48)* 6 and 47 *Population and intervention OR population and comparator (49)* 15 or 48 *Excluded comparator (50–54)* Trimethoprim-Sulfamethoxazole Combination/ Sulfamethoxazole.ab,ti. sulphamethoxazole.ab,ti. 50 or 51 or 52 49 not 53 *Search filter to identify RCTs (92–106)* randomized controlled trial.pt. controlled clinical trial.pt. randomized controlled trials/ random allocation/ double blind method/ single blind method/ clinical trial.pt. exp Clinical Trial/ (clin$ adj25 trial$).ti,ab. ((singl$ or doubl$ or trebl$ or tripl$) adj25 (blind$ or mask$)).ti,ab. placebos/ placebos.ti,ab. random.ti,ab. research design/ or/92-105 *(Population and Intervention OR population and comparator) AND RCT filter (107)* 54 and 106
**Search results**
The searches identified 978 citations, of which 958 were excluded by title or abstract. Twenty full papers were reviewed. Seven were excluded due to having no clinical outcome measure. Of the remaining 13 RCTs, 11 formed a connected network of evidence and were used in the network meta analysis (NMA). A total of 3983 participants were randomised across the trials, with the mean age across the trials ranging from 21 to 48 years.
**Evidence synthesis**
Evidence on clinical cure rates for the different regimens was synthesised by NMA using a random (treatment) effects model, with a logit link function to allow for heterogeneity in treatment effects between studies. All analyses were implemented in WinBUGS.^35^ Results of the NMA are reported in terms of the odds ratios and 95% credible intervals relative to fosfomycin 3 g, which was used as the reference intervention. Absolute estimates of clinical cure rates were estimated for each intervention by projecting the estimates of treatment effect (log OR) from the NMA onto the fosfomycin 3 g clinical cure rates.
